# Effect of Whey Supplementation on Circulating C-Reactive Protein: A Meta-Analysis of Randomized Controlled Trials

**DOI:** 10.3390/nu7021131

**Published:** 2015-02-09

**Authors:** Ling-Mei Zhou, Jia-Ying Xu, Chun-Ping Rao, Shufen Han, Zhongxiao Wan, Li-Qiang Qin

**Affiliations:** 1 Department of Nutrition and Food Hygiene, School of Public Health, Soochow University, 199 Renai Road, Suzhou 215123, China; E-Mails: zhoulingmei1112@sina.com (L.-M.Z.); sfhan@suda.edu.cn (S.H.); zhxwan@suda.edu.cn (Z.W.); 2 Key Laboratory of Radiation Biology, School of Radiation Medicine and Protection, Soochow University, 199 Renai Road, Suzhou 215123, China; E-Mail: xujiaying@suda.edu.cn; 3 Suzhou Health College, 28 Kehua Road, Suzhou 215009, China; E-Mail: rcping@sina.com; 4 Jiangsu Key Laboratory of Preventive and Translational Medicine for Geriatric Disease, Soochow University, 199 Renai Road, Suzhou 215123, China

**Keywords:** whey protein, C-reactive protein, randomized controlled trial, meta-analysis

## Abstract

Whey supplementation is beneficial for human health, possibly by reducing the circulating C-reactive protein (CRP) level, a sensitive marker of inflammation. Thus, a meta-analysis of randomized controlled trials was conducted to evaluate their relationship. A systematic literature search was conducted in July, 2014, to identify eligible studies. Either a fixed-effects model or a random-effects model was used to calculate pooled effects. The meta-analysis results of nine trials showed a slight, but no significant, reduction of 0.42 mg/L (95% CI −0.96, 0.13) in CRP level with the supplementation of whey protein and its derivates. Relatively high heterogeneity across studies was observed. Subgroup analyses showed that whey significantly lowered CRP by 0.72 mg/L (95% CI −0.97, −0.47) among trials with a daily whey dose ≥20 g/day and by 0.67 mg/L (95% CI −1.21, −0.14) among trials with baseline CRP ≥3 mg/L. Meta-regression analysis revealed that the baseline CRP level was a potential effect modifier of whey supplementation in reducing CRP. In conclusion, our meta-analysis did not find sufficient evidence that whey and its derivates elicited a beneficial effect in reducing circulating CRP. However, they may significantly reduce CRP among participants with highly supplemental doses or increased baseline CRP levels.

## 1. Introduction

Milk contains two high-quality proteins, namely casein and whey protein. Whey protein, which accounts for 20% of the total protein in bovine milk, has been a fairly useless liquid byproduct from cheese production for decades. However, whey protein represents a newly emerging class of biological substances with potential benefits for human health [1]. Our previous animal study found that whey protein improved insulin resistance and oxidative stress in rats fed with a high-fat diet (HFD) [2]. Our meta-analysis also indicated that the intake of milk protein-derived tripeptides resulted in a significant decrease in the blood pressure of prehypertensive and hypertensive patients [3]. It is well known that illnesses, such as cardiovascular disease (CVD), hypertension and type 2 diabetes mellitus (T2DM), are strongly related to inflammatory processes. On the other hand, chronic obstructive pulmonary disease (COPD) is a disease characterized by systemic inflammation. The common risk factors of COPD include cigarette smoking and air pollution [4]. Thus, it is likely feasible to prevent these diseases with the help of anti-inflammatory treatments [5,6,7]. C-reactive protein (CRP) is one of the most commonly determined inflammatory markers in clinical and epidemiologic studies. Several randomized controlled trials (RCTs) have assessed the level of circulating CRP in response to whey supplementation in subjects with different health conditions. However, the results of these trials are inconsistent, because of small sample sizes and quality that varied from low to high. Therefore, we conducted a meta-analysis of RCTs to examine whether or not the supplementation of whey and its derivates exhibits anti-inflammatory benefits. This meta-analysis also aimed to investigate the potential sources of heterogeneity across studies to elucidate current knowledge.

## 2. Methods

### 2.1. Literature Search

We make attempts to follow the Preferred Reporting Items for Systematic Reviews and Meta-Analysis (PRISMA) guidelines in the report of this meta-analysis [8] and registered our meta-analysis in PROSPERO (International prospective register of systemic reviews, http://www.crd.york.ac.uk/prospero; CRD42014015500). We conducted a systematic literature search of PubMed, the Web of Science and the Cochrane library through July, 2014, using the following search terms: “whey OR dairy OR milk” in combination with “inflammatory OR C-reactive protein OR CRP”. No restrictions were imposed. Reference lists were also reviewed. We did not contact the authors of the primary studies for additional information. We also did not try to take the unpublished studies into consideration.

### 2.2. Study Selection

Studies were selected in this meta-analysis if they: (1) were RCTs of whey protein or its derivates in adults (age ≥18 years old); (2) had an intervention duration ≥4 weeks; (3) had a control or a comparison group; and (4) included the net changes of CRP and their corresponding standard deviation (SD) or available data to calculate these values.

### 2.3. Data Extraction and Quality Assessment

We recorded the following characteristics of each study: first author’s name, publication year, study design, sample size, study period, whey protein type and daily dose. We also extracted the following participant characteristics: gender, mean age, body mass index (BMI), health condition, baseline CRP and change in CRP of each study. The data for CRP were converted into the same unit (mg/L). If more than one dose was administrated for supplementation, data from the highest dose were recorded. In the case of multiple publications with duplicated or overlapped data for the same trial, the article with more detailed information was selected. The Jadad score, a scale that ranges from 0 to 5 according to the descriptions of randomization, allocation concealment, blinding, withdrawal and availability of the intention-to-treat analysis, was used to measure the quality of each study [9].

### 2.4. Data Synthesis and Analysis

The net changes were calculated as the difference between the baseline and final values of CRP. For each study, the mean baseline CRP value was calculated by combining the mean values from the intervention and control groups, weighted by the number of participants. Studies with no reported SD values had their values imputed from standard errors, the confidence interval (CI) or *p*-values using a standard formula [10]. If only SD for the baseline and final values were provided, SD for the net changes were imputed according to the method of Follmann using a correlation coefficient of 0.5 [11].

The heterogeneity between the studies was tested using the Q test at the *p* < 0.10 level of significance and quantified by the *I*² statistic, which describes the inconsistency across studies [12]. In the presence of significant heterogeneity, the random-effects model was used to calculate the pooled effect size, and a meta-regression analysis was conduct to try to find out the possible sources of heterogeneity; otherwise, the fixed-effects model was applied [13].

We further conducted pre-specified subgroup analysis stratified by study duration, daily whey dose and baseline CRP level. We also performed a sensitivity analysis, in which a single trial was omitted each time and the effect size was recalculated to investigate its influence on the overall effect size. Potential publication bias was assessed using Begg’s funnel plots and Egger’s regression test [14]. All analyses were conducted by using STATA version 12.0 (StataCorp, College Station, TX, USA). *p* < 0.05 was considered statistically significant, except where otherwise specified.

## 3. Results

### 3.1. Search Results

**Figure 1:**
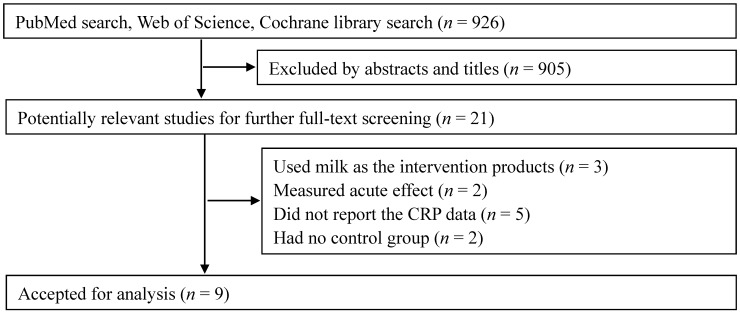
Flow chart of study selection.

The initial search found a total of 926 records, and 905 were excluded, because they were reviews/letters, animal experiments, observational studies or because the interventions were not relevant to our analysis. After a full-text review of the remaining 21 potentially relevant articles, 12 articles were further excluded because they used milk other than whey and its derivates as the intervention products (three articles), measured an acute effect (two articles), did not report the CRP data (five articles) or had no control group (two articles). Finally, nine RCTs were selected for our meta-analysis [15,16,17,18,19,20,21,22,23] (Figure 1).

### 3.2. Study Characteristics

The characteristics of the included trials are presented in Table 1. Nine trials were published from 2009 to 2014, in which two were conducted in Canada, Germany and the USA and one was conducted in Russia, Japan and Australia. A parallel design was used in all of the trials, and seven were double blinded. Sample sizes varied from 20 to 190 with a sum of 245 in the supplemental groups and 295 in the control groups. All trials enrolled men and women with mean ages ranging from 48.0 years to 77.3 years. Except one trial with healthy and two with overweight/obese adults, others involved patients with prehypertension, mild hypertension, COPD or metabolic syndrome. Three trials performed exercise training in the control and supplemental groups. Six trials excluded the participants using lipid-lowering drugs or related treatments [16,17,18,21,22,23]. Among nine trials, three trials expressed the outcome as high-sensitivity CRP (hsCRP) [15,17,22]. In these trials, only two trials used CRP as the primary endpoint [18,21]. Baseline CRP varied considerably from 0.4 mg/L to 7.6 mg/L with a median of 2.6 mg/L. The intervention duration lasted from four weeks to 36 weeks with a median of 12 weeks. In the nine trials, five trials used whey protein, two used whey protein peptides and the other two used fermented or hydrolyzed whey protein as the intervention. The intervention dose varied from 0.7 g to 60 g per day. Most control group received placebo or normal milk products without additional whey protein. In terms of study quality, all of the trials reported random allocation, but only three of the nine trials reported the details of sequence generation and allocation concealment. Dropout numbers and reasons were mentioned in all of the trials; in three trials, data were analyzed according to an intention-to-treat principle. For the Jadad score, one study had the highest score (= 5) and the other studies had scores of four and three.

### 3.3. Effect of Whey Protein on CRP

**Figure 2:**
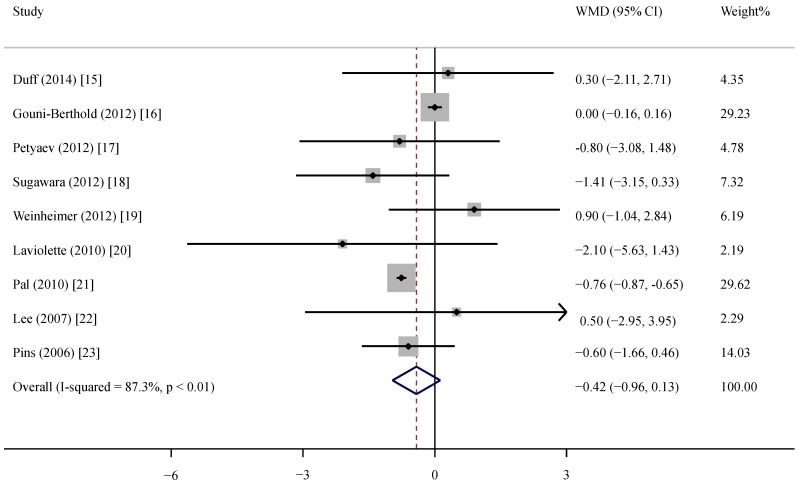
Meta-analysis of the effect of whey supplementation on circulating CRP level as compared with the control. WMD, weighted mean difference.

The supplementation of whey protein and its derivates was associated with an average net change in CRP ranging from −2.10 mg/L to 0.90 mg/L compared with the control group (Figure 2). CRP decreased in response to supplementation in five of the nine trials, but this reduction was statistically significant in only one trial. Considering the evidence of heterogeneity (*p* < 0.01, *I*^2^ = 87.3), we applied the random-effects model. The pooled effect of whey supplementation on circulating CRP level was −0.42 mg/L (95% CI, −0.96, 0.13) compared with that of the control group.

**Table 1:** Characteristic of the trials and participants in this meta-analysis.

Author	Year	Country	Study Design	Sample Size ^a^	Health Status	Male (%)	Age (year)	BMI (kg/m^2^)	Baseline CRP (mg/L)	CRP Assay	Duration (Weeks)	Intervention	Daily Dose (g)	Jadad Scores
Duff	2014	Canada	P, DB	21/19	Adults ^b^	37.5	59.5	NR	2.2	ELISA	8	Whey protein complex	38	4
Gouni-Berthold	2012	Germany	P, DB	88/92	Metabolic syndrome	52.8	53.4	31.1	0.4	Immunoturbidimetric latex highly-sensitive assay	12	Whey fermentation products	15.3	5
Petyaev	2012	Russia	P ^c^	10/10	Prehypertension	55.0	54.5	26.4	7.6	Commercially available kits	4	Whey protein isolate	0.7	3
Sugawara	2012	Japan	P, DB	17/14	COPD ^b^	93.5	77.3	NR	2.0	Latex turbidimetric immunoassay	12	Whey peptides	10	4
Weinheimer	2012	USA	P, DB	30/84	Overweight/obesity ^b^	40.4	48.0	30.0	3.2	COBAS Integra 400	36	Whey protein	60	3
Laviolette	2010	Canada	P, DB	12/10	COPD	63.6	65.3	28.2	4.0	Immunonephelometry	8	Pressurized whey	20	4
Pal	2010	Australia	P, SB	25/25	Overweight/obesity	14.0	48.5	31.3	3.8	Solid phase enzyme amplified sensitivity immunoassay	12	Whey protein isolate	54	3
Lee	2007	Germany	P, DB	27/26	Mild hypertension	56.7	51.6	27.9	2.3	Immunonephelometry	12	Whey peptides	3.0	4
Pins	2006	USA	P, DB	15/15	Prehypertension or stage 1 hypertension	46.7	46.1	29.0	2.6	Immunonephelometry	6	Hydrolyzed whey protein	20	3

P: parallel; SB: single blind; DB: double blind; NR: not reported; COPD: chronic obstructive pulmonary disease; ^a^ the sample size is expressed as intervention group/control group; ^b^ exercise training was simultaneously conducted; ^c^ no information about blinded design was provided.

### 3.4. Subgroup and Sensitivity Analyses

The results of subgroup analyses are presented in Table 2. Overall, whey supplementation produced a significantly greater CRP reduction among the trials at a whey dose ≥20 g/day (−0.72 mg/L, 95% CI −0.97, −0.47) and with a baseline CRP ≥3 mg/L (−0.67 mg/L, 95% CI −1.21, −0.14). In the sensitivity analyses, omitting the trial by Pal resulted in a small reduction of 0.02 mg/L (95% CI −0.18, 0.13), and omitting the trial by Gouni-Berthold resulted in a significant reduction of 0.75 mg/L (95% CI −0.86, −0.64). None of the other trials could substantially influence the pooled effect ranging from −0.34 mg/L (95% CI −0.90, 0.23) to −0.50 mg/L (95% CI −1.06, 0.06). Of note, when the trial of Pal or Gouni-Berthold was removed, no evidence of heterogeneity across the studies (*p* = 0.43, *I*^2^ = 0%; *p* = 0.63, *I*^2^ = 0%, respectively) was observed, suggesting that these two trials were the main sources of heterogeneity.

**Table 2:** Subgroup analyses according to study duration, daily whey supplementation and baseline CRP level.

Group	No.	Net Change (95% CI)	*p*	*P*_heterogeneity_	*I*^2^ (%)
Total	9	−0.42 (−0.96, 0.13)	0.20	<0.01	87.3
Study duration, week					
<12	4	−0.60 (−1.47, 0.26)	0.17	0.74	0
≥12	5	−0.30 (−1.02, 0.42)	0.41	<0.01	94.9
Whey dose, g/day					
<20	4	−0.10 (−0.69, 0.49)	0.74	0.32	14.1
≥20	5	−0.72 (−0.97, −0.47)	<0.01	0.39	3.8
CRP/hsCRP in study					
CRP	6	−0.75 (−0.85, −0.64)	<0.01	0.47	0
hsCRP	3	−0.32 (−0.99, 0.35)	0.35	0.16	45.2
Baseline CRP, mg/L					
<3	5	−0.06 (−0.46, 0.35)	0.79	0.34	11.8
≥3	4	−0.67 (−1.21, −0.14)	0.01	0.34	10.4

### 3.5. Meta-Regression Analysis

A meta-regression analysis was conducted to explore the sources of heterogeneity. To minimize the likelihood of false-positive results, we carefully selected a small number of covariates, including study duration, daily whey dose and baseline CRP. We found that baseline CRP was significantly associated with the effect estimate (*p* < 0.01) and accounted for 100% of the total between-study variation; by contrast, duration and dose were not associated with a net change in CRP (*p* = 0.25, 0.93). Therefore, baseline CRP was considered as a major source of heterogeneity among trials.

### 3.6. Publication Bias

Begg’s test suggested no evidence of publication bias for the outcomes (*p* = 0.18). Egger’s test also did not indicate the evidence of publication bias (*p* = 0.21).

## 4. Discussion

To the best of our knowledge, this study is the first meta-analysis of RCT to examine the effect of whey supplementation on circulating CRP level. Our primary result showed that supplemental whey protein and its derivates were insufficient to change the circulating CRP level. However, the supplementation produced a significant CRP reduction among participants with high supplemental doses or increased baseline CRP.

This meta-analysis followed the PRISMA guidelines and had a relatively high Jadad score. However, it was primarily limited by considerable heterogeneity across studies, which complicated the interpretation of our findings. This is not surprising, given the variation in study characteristics. In this meta-analysis, intervention included whey protein, hydrolyzed whey protein and whey peptides. Each component from them may function differently in terms of inflammatory response. The participants also included overweight/obese adults, as well as patients with hypertension, COPD or metabolic syndrome. Healthy status may differently influence the effect of whey protein on inflammatory response. In addition, genetic background or a gene-diet interaction may have been sources of heterogeneity across studies. Although we cannot further investigate the effect based on these characteristics because of the limited number of trials, the observed heterogeneity could be attributed to the two following trials, because heterogeneity disappeared after each of these trials was excluded in the sensitivity analyses. In the trial of Pal, the disparate results were likely due to the high daily dose of whey. This trial is the only one with a statistically significant CRP reduction [21]. In the trial of Gouni-Berthold, the baseline CRP level is only 0.4 mg/L. This value was the lowest among the trials. This low value may be partly attributable to the two months’ dietary and lifestyle recommendations before the intervention, because this healthy recommendation may decrease the CRP level. The CRP-reducing effect became statistically significant after this trial was excluded [16].

Subgroup analysis results indicated the influence of daily whey dose on the change in CRP. We found that CRP reduction was more pronounced when whey supplementation was ≥20 g/day, suggesting that whey quantity is an important factor affecting CRP responses. Weinheimer reviewed clinical trials and considered that ≥35 g of whey protein per day is possibly necessary to enhance the effect on metabolic health responses [19]. In theory, the consumption of any dairy products by adults results in an almost complete breakdown of protein to peptides and to the amino acid level. Indeed, the susceptibility of whey protein and its derivates to intestinal enzymes is a major problem that complicates the therapeutic use of such proteins [17]. Thus, a high dose of whey protein may be required to elicit a measurable biological effect in humans.

Subgroup analysis results also indicated a significantly larger reduction of CRP in subjects with increased CRP (≥3 mg/L) at baseline. This finding was also supported by meta-regression analysis that baseline CRP was a major source of heterogeneity among trials. This result is important, because more than 3 mg/L of CRP results in a high risk of future CVD events [5]. A decrease in CRP is also helpful to alleviate T2DM, because the CRP level is positively associated with T2DM incidence [24]. Obesity, which is an established risk factor for CVD and T2DM, has been associated with elevated levels of CRP [25]. Thus, our finding is useful, because intervention could be effective in individuals who most needed this treatment.

In the subgroup analysis, a significant decrease of CRP was found in CRP and not in hsCRP. However, we should mention that hsCRP is the same kind of protein as CRP. CRP is called hsCRP when a highly-sensitive assay is used. In fact, hsCRP assays were commonly available after 2000 [26]. Some researchers still used the term CRP in the study, even if a highly-sensitive assay were used. Thus, the difference between CRP and hsCRP should be interpreted with caution because of a lack of unified nomenclature used in these studies.

Despite the insufficient evidence provided in the present study, whey protein possibly exhibited a potential CRP-lowering effect. CRP is principally induced by interleukin (IL)-6 and IL-8 via a mechanism involving the activation of the nuclear factor kappa B pathway, which is a key regulator of pro-inflammatory mediator synthesis [27,28,29]. Intense exercise generally induces increased levels of pro-inflammatory mediators [30]. The consumption of cake consisting of whey protein significantly reduces the exercise-increased CRP and IL-6 by 46% and 50%, respectively [31]. Cystic fibrosis is characterized by chronic pulmonary inflammation. Pressurized whey supplementation significantly decreases the increased CRP and IL-8 levels in these patients [32]. These two trials were excluded in the meta-analysis because of the acute intervention in the former and the lack of a control group in the latter. The effects of whey protein on inflammation are not limited to CRP. Several RCTs used other inflammatory markers, such as IL-6, IL-8 and TNF-α. For example, Sugawara found that circulating levels of IL-6, IL-8 and TNF-α in the patients with COPD significantly decreased after whey intervention compared with those in the control group [18]. However, no significant change in IL-6 and TNF-α was observed in overweight individuals between the whey group and control group [21].

Inflammation status and oxidative stress phenomena are narrowly interacting in disorders, such as obesity, CVD and T2DM [33]. Whey protein is rich in cysteine, which can increase the synthesis of glutathione, a crucial intracellular antioxidant [34]. In one trial included in this meta-analysis, the change in glutathione and CRP levels after pressurized whey protein was consumed by COPD patients was not observed [18]. However, another trial found that the pressurized whey protein supplementation of 45 g/day in healthy adults increased lymphocyte glutathione by 24% [35]. Likewise, our animal study observed that the plasma glutathione level was significantly higher in rats fed with HFD adding 15% whey protein than HFD-only fed rats [2]. A further *in vitro* study demonstrated that 6.24 mg/mL of whey protein increased the glutathione level by 138% in C2C12 muscle cells that induced oxidative stress by *tert*-butyl hydroperoxide [36]. Whey protein contains a high level of other essential amino acids, such as leucine. An animal study from our laboratory also confirmed that 1.6% leucine supplementation with 15% whey protein significantly enhanced the antioxidant capacity in non-obese insulin resistant rats [2]. In a clinical trial with overweight or obese subjects, administration of nutraceutical containing 2.25 g of leucine per day for four weeks significantly reduced oxidative and inflammatory biomarkers (TNF-α, CRP) and increased the anti-inflammatory marker, adiponectin [37]. Furthermore, milk-derived bioactive peptides exert beneficial effects on the prevention and treatment of chronic metabolic diseases via multiple mechanisms, such as regulation of insulin resistance and blood pressure, affecting the oxidative stress levels and alteration of the lipid profiles [3,38].

Finally, evidence from recent years also suggests that milk casein *per se* can actively affect the inflammatory process with inconsistent findings. Aihara found that oral administration of milk casein-derived tripeptide Val-Pro-Pro for 10 weeks exerts an anti-inflammatory effect on the adipose tissue of HFD-fed mice [39]. Hirota reported that casein hydrolysate containing Val-Pro-Pro and Ile-Pro-Pro supplementation in subjects with mild hypertension significantly reduced circulating TNF-α levels, although no alteration in circulating CRP level was found [40]. Similarly, no alterations in circulating CRP levels were observed in overweight adolescents supplemented with casein protein for a total of 12 week [41]. Therefore, more trials are required to further investigate the relationship between casein intake and inflammatory biomarkers.

## 5. Conclusions

Our findings did not support the overall favorable effect of whey protein on circulating CRP level, based on the current evidence. However, the supplementation of whey protein and its derivates may produce a significant reduction in CRP among participants supplemented with a high dose of whey proteins or increased baseline CRP level. Although we successfully identified baseline CRP concentration as a potential effect modifier and major contributor to the overall between-study variation in meta-regression analysis, our results should be interpreted with caution because of the evidence of heterogeneity.
